# Supplementary material for: Development and Validation of Risk Prediction Models for Colorectal Cancer in Patients with Symptoms
Source: J Pers Med. 2023 Jun 29;13(7):1065. doi: 10.3390/jpm13071065 (PMC10381199; doi:10.3390/jpm13071065)
Supplement: Supplementary file 1 [file jpm-13-01065-s001.zip › jpm-2434092-supplementary.pdf]

Supplementary Material

Table S1: CRC SNPs used for the generation of polygenic risk score

Table S2: $wGRS_{137}$, $wGRS_{163}$, $wGRS_{202}$ comparison

Table S3: Risk prediction models for CRC in patients with symptoms

Table S4: Comparison of CRC cases in SOCCS (n=1649) and LABSS (n=37)

Table S5: Age-CRC restricted cubic splines

Table S6: BMI-CRC restricted cubic splines

Table S7: $wGRS_{202}$-CRC restricted cubic splines

Table S8: Model C and model E comparison

Table S9: Summary of models A-D formula

Table S10: CRC risk prediction models that incorporated genetic predictors

Table S11: Methods for variable selection in the development of the final prediction model

Table S12: Random forest model F and model G comparison

Figure S1: Flow chart for $wGRS_{137}$, $wGRS_{163}$, $wGRS_{202}$

Figure S2: TRIPOD checklist

Figure S3: Plot-association between age and risk of CRC

Figure S4: Plot-association between BMI and risk of CRC

Figure S5: Plot-association between $wGRS_{202}$ and risk of CRC

Figure S6: Restricted cubic splines fit age with CRC risk

Figure S7: Restricted cubic splines fit BMI with CRC risk

Figure S8: Restricted cubic splines fit $wGRS_{202}$ with CRC risk

Figure S9: ROC curves- $wGRS_{137}$, $wGRS_{163}$, $wGRS_{202}$ comparison

Figure S10: Calibration curves- $wGRS_{137}$, $wGRS_{163}$, $wGRS_{202}$ comparison

Figure S11: Random forest parameters tuning: mtry versus OOB error

Figure S12: Model F_ Plot of OOB errors against number of trees

Figure S13: Model G_ Plot of OOB errors against number of trees

Table S1: CRC SNPs used for the generation of polygenic risk score

SNP_Ldproxy	Chr	Position	Allele_A	Allele_B	R^2	SNP/Ldproxpy	Coefficient (beta) extracted from meta-GWAS
rs2807367	1	22503282	G	C	NA	SNP	-0.03581
rs34963268	1	22710877	G	C	NA	SNP	-0.05861
rs28428561	1	38434306	G	A	0.8348	proxy	0.053686
rs200394259	1	55268836	CTTACA	C	0.5333	proxy	0.04143
rs7542665	1	62673037	T	C	NA	SNP	0.002712
rs3124454	1	71040166	G	T	NA	SNP	-0.04427
rs6660031	1	110365045	G	A	NA	SNP	0.029928
rs5028523	1	172864224	A	G	NA	SNP	-0.04043
rs10911251	1	183081194	A	C	0.9352	proxy	-0.0728
rs12137232	1	201885446	G	T	NA	SNP	-0.03853
rs896319	1	205205651	G	T	1	proxy	-0.06998
rs6691170	1	222045446	G	T	NA	SNP	0.056924
rs2078095	1	240408346	A	G	NA	SNP	0.042447
rs7606562	2	48686695	T	A	NA	SNP	-0.00419
rs11885030	2	98755785	G	A	0.0479	proxy	0.001765
rs1446585	2	136407479	A	G	NA	SNP	0.039735
rs842076	2	159818178	C	T	0.4158	proxy	-0.02816
rs4668039	2	169025379	A	G	1	proxy	0.038438
rs1455352	2	199485958	T	C	0.2775	proxy	0.032473
rs4675253	2	199888532	C	G	NA	SNP	0.063099
rs3731861	2	219191256	T	C	NA	SNP	-0.06418
rs35149869	3	37029108	T	T	1	proxy	0.019592
rs116080590	3	40955671	C	A	0.0424	proxy	0.116445
rs2001732	3	52880740	C	T	NA	SNP	-0.07592
rs2581817	3	53071797	G	C	NA	SNP	-0.0528
rs9826722	3	64316392	T	G	0.1531	proxy	0.020901
rs7623129	3	64624426	C	T	1	proxy	-0.03323
rs67550176	3	66444330	T	C	NA	SNP	0.048444
rs13086367	3	112903888	A	G	NA	SNP	-0.05269
rs138091542	3	112904785	C	T	0.1602	proxy	-0.07761
rs12635946	3	112916918	C	T	NA	SNP	-0.05528
rs10049390	3	133701119	G	A	NA	SNP	0.05287
rs113569514	3	133748789	T	C	NA	SNP	-0.06663
rs10936599	3	169492101	C	T	NA	SNP	-0.05259
rs34880024	4	94957435	T	C	0.6928	proxy	0.043526

rs7663401	4	106128954	C	T	1	proxy	0.056482
rs2388976	4	115502406	G	A	1	proxy	0.03638
rs4835192	4	145738397	A	T	0.2095	proxy	0.046627
rs12641861	4	151660362	T	G	0.7732	proxy	0.026339
rs1426947	4	175420523	T	C	NA	SNP	-0.03669
rs77776598	5	1240998	T	C	NA	SNP	0.139631
rs2735940	5	1296486	A	G	NA	SNP	0.086259
rs55810369	5	40090784	C	T	NA	SNP	-0.05252
rs1445012	5	40282106	G	C	NA	SNP	0.094153
rs3930345	5	82881255	C	T	NA	SNP	-0.0487
rs12659017	5	125988175	G	A	NA	SNP	0.002367
rs647161	5	134499092	C	A	NA	SNP	0.041701
rs2302274	5	149546426	A	G	NA	SNP	0.047889
rs472959	5	172324558	G	A	NA	SNP	0.035336
rs1294437	6	6749789	C	T	NA	SNP	-0.03444
rs41302867	6	7240876	G	A	0.4589	proxy	-0.06979
rs2070699	6	12292772	G	T	NA	SNP	0.054108
rs209142	6	28862617	G	C	NA	SNP	0.037326
rs1476570	6	29809860	G	A	NA	SNP	-0.00267
rs116353863	6	31010185	T	C	NA	SNP	0.164692
rs2517448	6	31062667	T	C	NA	SNP	0.043293
rs62401893	6	31317361	G	A	NA	SNP	0.104554
rs2516452	6	31427095	A	G	NA	SNP	-0.07378
rs3830041	6	32191339	C	T	NA	SNP	0.007388
rs112226346	6	32586732	G	C	NA	SNP	0.084463
rs16878812	6	35569562	A	G	NA	SNP	-0.08758
rs9470361	6	36623379	G	A	NA	SNP	0.06533
rs9471632	6	41680666	T	C	0.3003	proxy	0.013488
rs9472532	6	45605345	C	A	0.9465	proxy	0.066799
rs2208605	6	55572187	T	C	0.7163	proxy	0.042135
rs62404966	6	55712124	C	T	NA	SNP	-0.06557
rs6912214	6	55721302	T	C	NA	SNP	-0.04215
rs6928864	6	105966894	C	A	NA	SNP	-0.11653
rs145997965	6	106482613	T	C	NA	SNP	0.189455
rs6911915	6	117809031	T	C	NA	SNP	0.044014
rs151127921	6	133993925	C	T	NA	SNP	0.159257
rs1182197	7	2863289	A	C	NA	SNP	-0.03785

rs7810512	7	45150331	A	C	NA	SNP	-0.04833
rs80077929	7	46094089	C	T	NA	SNP	0.060381
rs4236382	7	46889411	G	A	NA	SNP	0.045892
rs6948177	7	47510741	A	G	NA	SNP	0.06087
rs12539962	7	73167259	C	T	NA	SNP	-0.04617
rs2527927	7	99477426	G	A	NA	SNP	-0.04186
rs157901	7	130618123	C	T	0.1643	proxy	-0.03357
rs59076710	8	23634311	G	T	0.3184	proxy	-0.01797
rs826732	8	59742639	G	C	NA	SNP	0.034161
rs2450115	8	117624093	T	C	NA	SNP	-0.07224
rs16892766	8	117630683	A	C	NA	SNP	0.201008
rs117904730	8	117682626	T	C	0.5864	proxy	-0.09809
rs6983267	8	128413305	G	T	NA	SNP	-0.151
rs7013278	8	128414892	T	C	NA	SNP	-0.14629
rs10110900	8	128571059	G	A	0.4538	proxy	-0.02897
rs7859362	9	22105927	T	C	NA	SNP	-0.06085
rs12001437	9	34074476	T	C	0.299	proxy	0.01656
rs13288865	9	101682572	T	G	1	proxy	-0.06488
rs10978941	9	110373819	C	T	NA	SNP	-0.0575
rs10817106	9	113654654	G	C	NA	SNP	0.052647
rs7038489	9	136682468	C	T	NA	SNP	-0.0756
rs11789898	9	136925663	G	T	NA	SNP	0.049837
rs12217641	10	8663875	C	T	NA	SNP	0.008249
rs11255841	10	8739580	T	A	NA	SNP	-0.09871
rs11007250	10	29098096	C	G	0.4246	proxy	0.043471
rs1773860	10	29291556	C	T	NA	SNP	0.037151
rs10821905	10	52646093	G	A	NA	SNP	-0.06695
rs704017	10	80819132	A	G	NA	SNP	0.086584
rs1782645	10	81048611	C	T	NA	SNP	0.047229
rs11595126	10	91428283	G	A	0.9331	proxy	0.042329
rs6584283	10	101290301	T	C	NA	SNP	0.021914
rs35564340	10	101344263	G	A	NA	SNP	0.094575
rs4919687	10	104595248	G	A	NA	SNP	-0.01828
rs12241008	10	114280702	T	C	NA	SNP	0.09986
rs11196201	10	114803307	A	T	0.6456	proxy	0.043121
rs11042725	11	10325325	C	A	0.9569	proxy	0.0349
rs174537	11	61552680	G	T	NA	SNP	-0.05374

rs10751097	11	69938433	A	G	NA	SNP	-0.04777
rs11236187	11	74364566	A	C	NA	SNP	0.078194
rs7946853	11	74409077	T	C	NA	SNP	0.02892
rs117042741	11	74628743	C	T	NA	SNP	-0.22612
rs55864876	11	100717136	G	A	NA	SNP	-0.06769
rs2155065	11	101616011	C	T	NA	SNP	-0.04081
rs3087967	11	111156836	T	C	NA	SNP	-0.12654
rs497916	11	118758089	C	T	NA	SNP	0.037147
rs10774214	12	4368352	T	C	NA	SNP	-0.03247
rs3217861	12	4399054	C	T	0.2761	proxy	0.056817
rs3217874	12	4400808	C	T	NA	SNP	0.06869
rs187299794	12	5941363	C	T	0.0503	proxy	-0.05583
rs4764488	12	6332910	A	G	0.0859	proxy	-0.00888
rs2730985	12	43130624	A	G	NA	SNP	0.044797
rs11169572	12	51216890	T	C	NA	SNP	0.086013
rs715948	12	57532982	T	C	0.6274	proxy	0.048498
rs7297628	12	64404555	T	C	NA	SNP	-0.03548
rs11178634	12	71518329	G	T	NA	SNP	-0.04564
rs11108175	12	96050887	A	G	NA	SNP	-0.04837
rs653178	12	112007756	C	T	NA	SNP	0.075734
rs9634162	12	115098094	G	A	NA	SNP	0.03684
rs7300312	12	115890922	T	C	NA	SNP	0.065566
rs7299936	12	115934000	G	A	NA	SNP	0.053016
rs73208120	12	117747590	T	G	NA	SNP	0.054895
rs116964464	13	27543193	C	T	NA	SNP	0.101886
rs10161980	13	34093518	C	G	NA	SNP	-0.05268
rs9603116	13	37493701	A	G	0.4068	proxy	-0.02748
rs45597035	13	73649152	A	G	NA	SNP	-0.06143
rs78341008	13	73791554	T	C	NA	SNP	0.107452
rs1886450	13	73986628	A	G	NA	SNP	0.032069
rs79665926	13	78578231	G	T	0.8885	proxy	-0.05245
rs1078563	13	110352851	G	C	NA	SNP	-0.04052
rs4600332	13	111078872	A	G	NA	SNP	0.056484
rs28611105	14	51359658	T	G	NA	SNP	0.050232
rs1497077	14	52491655	T	C	NA	SNP	0.039419
rs1951864	14	54369299	G	A	NA	SNP	0.040672
rs35107139	14	54419106	A	C	NA	SNP	0.089335

rs4901473	14	54445157	G	A	NA	SNP	-0.06014
rs17094983	14	59189361	G	A	NA	SNP	-0.06365
rs8020436	14	59208437	G	A	NA	SNP	0.060355
rs61975764	14	93014929	G	A	NA	SNP	-0.02575
rs8014920	14	99785345	G	A	0.2356	proxy	0.030242
rs1554865	15	32999806	C	T	NA	SNP	-0.13772
rs1919364	15	33009574	C	G	NA	SNP	0.074368
rs17816465	15	33156386	G	A	NA	SNP	0.078246
rs3809570	15	67000117	C	A	NA	SNP	-0.05398
rs28647290	15	67405582	G	A	0.2646	proxy	0.002968
rs77148098	15	68075698	G	A	NA	SNP	0.059136
rs78473344	15	72395312	T	G	0.0971	proxy	0.042147
rs79973254	15	91191441	T	A	0.8514	proxy	0.075035
rs4783565	16	68750190	G	A	0.9739	proxy	-0.05073
rs4556797	16	80037987	A	G	0.2378	proxy	0.036633
rs76702092	16	86257480	C	G	0.1156	proxy	-0.02489
rs7200646	16	86335351	C	T	0.501	proxy	-0.0421
rs62042090	16	86703949	C	T	NA	SNP	0.05934
rs142192012	17	594005	C	T	0.4972	proxy	-0.0426
rs4968127	17	809643	G	A	NA	SNP	-0.05888
rs1806551	17	831873	T	C	0.0552	proxy	0.005076
rs9899841	17	10714548	C	T	0.3894	proxy	0.038615
rs983318	17	70413253	G	A	NA	SNP	0.057813
rs3088242	17	80939495	T	A	0.0612	proxy	-0.0051
rs112795617	18	3166445	T	A	0.0781	proxy	0.020757
rs2337113	18	46452327	A	G	NA	SNP	-0.16435
rs3752231	19	1043638	C	T	0.0424	proxy	-0.0188
rs10409772	19	5840926	C	A	NA	SNP	0.077136
rs66850493	19	16428747	G	A	0.889	proxy	0.08379
rs28840750	19	33519927	T	G	NA	SNP	-0.19119
rs1800469	19	41860296	A	G	NA	SNP	0.053034
rs12979278	19	49218602	C	T	NA	SNP	0.036353
rs7253433	19	59041723	G	A	0.12	proxy	0.002448
rs966816	20	6376481	G	A	NA	SNP	-0.09793
rs994308	20	6603622	C	T	NA	SNP	-0.07663
rs4140509	20	6679139	C	T	0.0562	proxy	0.003774
rs235755	20	6767119	G	C	0.0619	proxy	0.006553

rs2423279	20	7812350	T	C	NA	SNP	0.065712
rs55695988	20	33090447	G	A	0.073	proxy	-0.00352
rs6031256	20	42583617	A	G	0.0675	proxy	-0.00486
rs11482045	20	42714142	C	C	0.0338	proxy	-0.00537
rs6066825	20	47340117	A	G	NA	SNP	-0.07561
rs6012915	20	48986567	C	T	NA	SNP	-0.06523
rs6095946	20	49060191	C	T	NA	SNP	-0.06491
rs6091189	20	49256285	C	T	NA	SNP	0.059205
rs6014965	20	55831203	A	G	NA	SNP	-0.04661
rs8121252	20	57476809	T	C	0.7985	proxy	-0.00191
rs1741640	20	60932414	T	C	NA	SNP	0.127997
rs2738783	20	62308612	T	G	NA	SNP	-0.06247
rs8127251	21	47797626	G	A	0.1306	proxy	-0.0218
rs4616575	22	29406076	T	G	NA	SNP	-0.04051
rs4820371	22	39659088	T	C	0.4601	proxy	0.026811
rs5759256	22	43680239	T	C	0.1582	proxy	0.003187
rs20195	22	43857328	C	T	0.7549	proxy	0.000142
rs9614460	22	45745229	T	G	NA	SNP	0.048501
rs7410394	22	46455794	A	G	0.0936	proxy	-0.01033

[Variants: 202 SNPs used for calculating wGRS; CHR: cell cycle genes homology region; Position: position on a chromosome where a particular SNP is located; ACGT is an acronym for the four types of bases found in a DNA molecule: adenine (A), cytosine (C), guanine (G), and thymine (T); Coefficient (beta) extracted from the meta-GWAS published beta values of 202 SNPs. wGRS is the number of risk alleles carried at each genetic variant SNP is summed, weighted by its effect size (beta coefficient).]

Table S2: $wGRS_{137}$, $wGRS_{163}$, $wGRS_{202}$ comparison

Model	Method	Case	Control	λ	Predictors	Intercept	Coefficient	OR (95% CI)	P-value	R^2	Brier	AIC	BIC	C-statistic	Corrected C-statistic	HL (P-value)
					wGRS137		0.7297	2.07 (1.68-2.56)	1.10E-11							
					Age		0.041	1.04 (1.03-1.05)	3.08E-29							
Model A1	LASSO	1686	963	0.025739	Sex	-1.1731	0.366	1.44 (1.21-1.72)	5.61E-05	0.264	0.184	2917.531	2952.822	0.766 (0.747-0.785)	0.764	0.1612
					Change of bowel habit		-1.2387	0.29 (0.24-0.35)	8.99E-39							
					Abdominal pain		-0.6802	0.51 (0.42-0.61)	6.28E-12							
					wGRS163		0.7532	2.12 (1.73-2.62)	1.26E-12							
					Age		0.0409	1.04 (1.03-1.05)	3.89E-29							
Model A2	LASSO	1686	963	0.025739	Sex	-1.2633	0.3632	1.44 (1.20-1.72)	6.50E-05	0.266	0.183	2912.991	2948.282	0.766 (0.748-0.785)	0.765	0.06302
					Change of bowel habit		-1.2405	0.29 (0.24-0.35)	8.14E-39							
					Abdominal pain		-0.6764	0.51 (0.42-0.62)	8.63E-12							
					wGRS202		0.7612	2.14 (1.74-2.64)	5.31E-13							
					Age		0.041	1.04 (1.03-1.05)	3.53E-29							
Model A3	LASSO	1686	963	0.025739	Sex	-1.3030	0.3611	1.43 (1.20-1.72)	7.19E-05	0.266	0.183	2911.234	2946.526	0.767 (0.748-0.786)	0.765	0.02385
					Change of bowel habit		-1.2411	0.29 (0.24-0.35)	8.06E-39							
					Abdominal pain		-0.6784	0.51 (0.42-0.62)	7.65E-12							
					Age		0.0401	1.04 (1.03-1.05)	1.06E-28							
					Sex		0.369	1.45 (1.21-1.73)	4.09E-05							
Model B	LASSO	1686	963	0.031003	Change of bowel habit	-1.2124	-1.2411	0.29 (0.24-0.35)	1.34E-39	0.244	0.188	2962.84	2992.25	0.754 (0.735-0.774)	0.753	0.7107
					Abdominal pain		-0.702	0.50 (0.41-0.60)	7.77E-13							
					wGRS137		0.7291	2.07 (1.68-2.56)	1.37E-11							
					Age		0.041	1.04 (1.03-1.05)	2.27E-28							
					Sex		0.3682	1.45 (1.21-1.73)	5.46E-05							
					BMI		-0.0196	0.98 (0.96-1.00)	0.017999							
Model C1	full model	1686	963	NA	Family history	-0.6360	-0.0083	0.99 (0.78-1.26)	0.946053	0.266	0.183	2921.385	2986.087	0.766 (0.748-0.785)	0.763	0.03683
					Change of bowel habit		-1.2596	0.28 (0.23-0.34)	8.30E-37							
					Rectal bleeding		0.0422	1.04 (0.86-1.27)	0.670474							
					Weight loss		-0.0107	0.99 (0.78-1.26)	0.930695							
					Anaemia		-0.0526	0.95 (0.74-1.22)	0.681661							

					Abdominal pain		-0.6802	0.51 (0.42-0.62)	1.31E-11							
Model C2	full model	1686	963	NA	wGRS163	-0.7315	0.7517	2.12 (1.72-2.62)	1.67E-12	0.268	0.183	2916.983	2981.684	0.767 (0.748-0.786)	0.763	0.007456
					Age		0.041	1.04 (1.03-1.05)	2.90E-28							
					Sex		0.3653	1.44 (1.20-1.72)	6.33E-05							
					BMI		-0.0193	0.98 (0.97-1.00)	0.01946							
					Family history		-0.0026	1.00 (0.78-1.27)	0.983021							
					Change of bowel habit		-1.2616	0.28 (0.23-0.34)	7.45E-37							
					Rectal bleeding		0.0399	1.04 (0.86-1.27)	0.688164							
					Weight loss		-0.0084	0.99 (0.78-1.26)	0.945661							
					Anaemia		-0.0547	0.95 (0.74-1.22)	0.669637							
					Abdominal pain		-0.6771	0.51 (0.42-0.62)	1.70E-11							
Model C3	full model	1686	963	NA	wGRS202	-0.7679	0.7603	2.14 (1.74-2.64)	6.91E-13	0.269	0.183	2915.181	2979.883	0.767 (0.749-0.786)	0.764	0.01758
					Age		0.041	1.04 (1.03-1.05)	2.65E-28							
					Sex		0.3631	1.44 (1.20-1.72)	7.05E-05							
					BMI		-0.0195	0.98 (0.96-1.00)	0.018733							
					Family history		-0.0024	1.00 (0.78-1.27)	0.984612							
					Change of bowel habit		-1.2616	0.28 (0.23-0.34)	7.68E-37							
					Rectal bleeding		0.0402	1.04 (0.86-1.27)	0.685827							
					Weight loss		-0.0112	0.99 (0.78-1.26)	0.927834							
					Anaemia		-0.0531	0.95 (0.74-1.22)	0.678512							
					Abdominal pain		-0.6786	0.51 (0.42-0.63)	1.55E-11							
Model D	full model	1686	963	NA	Age	-0.7170	0.0404	1.04 (1.03-1.05)	4.12E-28	0.247	0.187	2966.24	3025.059	0.755 (0.736-0.775)	0.752	0.4278
					Sex		0.3714	1.45 (1.21-1.73)	3.94E-05							
					BMI		-0.0191	0.98 (0.97-1.00)	0.019995							
					Family history		0.0349	1.04 (0.82-1.32)	0.773835							
					Change of bowel habit		-1.2667	0.28 (0.23-0.34)	7.07E-38							
					Rectal bleeding		0.0734	1.08 (0.89-1.31)	0.45527							
					Weight loss		-0.0053	0.99 (0.78-1.27)	0.965515							
					Anaemia		-0.0661	0.94 (0.73-1.20)	0.602107							
					Abdominal pain		-0.6999	0.50 (0.41-0.60)	2.03E-12							

Table S3: Risk prediction models for CRC in patients with symptoms

Author, year	Country	Study design	Population	Outcome	Sample size (development /validation)	Predictors	Model performance (95%CI)	Model presentation	Internal validation
Fijten, 1995	The Netherlands	D	patients with overt rectal bleeding or a history of visible rectal blood loss in previous 3 months	CRC	269	age<br>change of bowel habit<br>rectal bleeding	AUC: 0.97	risk score	NA
Adelstein, 2010	Australia	D	patients >18 years scheduled for colonoscopy	CRC	8204	age<br>sex<br>rectal bleeding<br>abdominal pain<br>anaemia<br>mucous<br>history of diverticular disease<br>NSAID or aspirin use	AUC: 0.85	risk score	NA
Adelstein, 2011	Australia	D	patients >18 years scheduled for colonoscopy	CRC	8204	age<br>sex<br>smoking<br>education level<br>rectal bleeding<br>anaemia<br>mucous<br>fatigue<br>history of diverticular disease<br>irritable bowel syndrome<br>NSAID or aspirin use	AUC: 0.83	risk score	NA
Hurst, 2007	UK	D	patients >18 years referred to a colorectal clinic	CRC	300	age<br>sex<br>smoking<br>abdominal pain<br>weight loss<br>sMMP-9	sensitivity: 0.779;<br>specificity: 0.771	formula	NA
Lam, 2002	China	D	patients attending surgical department for rectal bleeding	CRC	194	age<br>rectal bleeding<br>mucous	sensitivity: 0.731;<br>specificity: 0.628	risk score	NA

Mahadavan,2011	UK	D	patients >40 years referred to a CRC surgical clinic	CRC	714	age<br>sex<br>rectal bleeding<br>FOBT<br>mean red cell volume (fl)<br>carcino-embryonic antigen (µg/l)<br>DNA (µg/ml)	AUC: 0.84 (0.78–0.90)	risk score	NA
Hamilton, 2005	UK	D	patients >40 years from Exeter Primary Care Trust	CRC	2093	rectal bleeding<br>diarrhoea<br>abdominal pain<br>weight loss<br>constipation<br>tenderness on palpation of abdomen<br>haemoglobin <13 g/dl<br>blood sugar >10 mmol/l<br>FOBT	positive predictive values	NA	NA
Hamilton, 2009	UK	D	patients >30 years from Health Improvement Network (THIN)	CRC	43791	rectal bleeding<br>change in bowel habit<br>diarrhoea<br>abdominal pain<br>weight loss<br>constipation<br>haemoglobin (g/dl)<br>mean red cell volume (fl)	positive predictive values	NA	NA
Bjerregaard, 2007	Denmark	D	outpatients aged >40 years with symptoms consistent with CRC	CRC	2172	age<br>sex<br>anaemia<br>weight loss<br>abdominal pain<br>rectal bleeding<br>rectal mucous<br>change in frequency of bowel movements<br>change in stool consistency<br>insufficient rectal emptying	NA	NA	NA
Thompson, 2007	UK	D	patients with lower gastrointestinal symptoms referred to a surgical outpatient clinic	CRC	8529	age<br>change in bowel habit<br>rectal bleeding<br>perianal symptoms	ROC curve	NA	NA

Hippisley-Cox, 2012	UK	D+IV	30–84 years patients registered with practices	CRC	2351052/1236601	age<br>alcohol<br>rectal bleeding<br>change in bowel habit<br>abdominal pain<br>weight loss<br>loss of appetite<br>haemoglobin (g/dl)<br>family history of gastrointestinal cancer	female: AUC 0.89 (0.88-0.90)<br><br>male: AUC 0.91 (0.90-0.91)	risk score	split sample
Li, 2018*	China	D+IV	community residents aged 60 to 74 years participated in screening program	CRC	890235	age<br>sex<br>education level<br>occupation<br>diarrhoea<br>constipation<br>colon mucus and bleeding<br>gallbladder disease<br>stressful life event<br>CRC family history<br>FIT	AUC: 0.838 (0.817–0.860)<br><br>calibration (P=0.1549)	risk score	bootstrap
Alatise, 2018*	Africa	D+IV	patients with rectal bleeding who underwent complete colonoscopy	CRC	217/145	age<br>sex<br>BMI<br>change of bowel habit<br>alternating<br>constipation<br>diarrhoea<br>pellet-like stool<br>weight loss<br>rectal bleeding<br>internal hemorrhoids<br>diverticulum	AUC: 0.875	risk score	random split
Chen, 2021*	China	D+IV	residents participated in MJ Health Management Institution (MJ) for self-paying medical screening	CRC	234044	age<br>sex<br>smoking<br>drinking<br>physical activity<br>fruit and vegetables<br>BMI<br>family history<br>black colour stool<br>change of bowel habit<br>anaemia	total colon: AUC: 0.73 (0.71–0.74)<br><br>proximal colon: AUC: 0.74 (0.71–0.76)<br><br>distal colon: AUC: 0.73 (0.70–0.76)<br><br>rectum: AUC: 0.71 (0.69–0.73)	risk score	random split

Chen, 2021*	China	D+IV	residents participated in MJ Health Management Institution (MJ) for self-paying medical screening	CRC	234044	age<br>sex<br>smoking<br>drinking<br>physical activity<br>fruit and vegetables<br>BMI<br>family history<br>CEA<br>CRP<br>FIT<br>hypertension<br>diabetes<br>black colour stool<br>change of bowel habit<br>anaemia	total colon: AUC: 0.83 (0.81–0.85)<br><br>proximal colon: AUC: 0.83 (0.80–0.86)<br><br>distal colon: AUC: 0.86 (0.84–0.88)<br><br>rectum:<br>AUC: 0.80 (0.78–0.83)	risk score	random split
Selvachandran, 2002	UK	D+EV	patients with distal colonic symptoms, referred by general practitioners	CRC	2268	age<br>sex<br>family history<br>rectal bleeding<br>change in bowel habit<br>weight loss<br>abdominal pain<br>loss of appetite<br>tenesmus<br>urgency<br>incomplete emptying<br>perianal symptoms<br>tiredness	internal AUC: 0.859<br><br>external AUC: 0.789	risk score	NA
Cubiella, 2016*	Spain	D+EV	patients with gastrointestinal symptoms referred for colonoscopy from primary and secondary health care	CRC	1572/1481	age<br>sex<br>faecal haemoglobin<br>blood haemoglobin<br>carcinoembryonic antigen<br>acetylsalicylic acid treatment<br>previous colonoscopy<br>benign anorectal lesion<br>rectal bleeding<br>change in bowel habit<br>rectal mass	internal AUC: 0.92 (0.91-0.93)<br><br>external AUC: 0.92 (0.90-0.94)	risk score	NA
Marshall, 2011	UK	D+EV	cases were patients aged 30 years or older with a diagnosis of CRC, seven controls per case, matched for practice, sex and age	CRC	43791/2093	haemoglobin<br>MCV<br>rectal bleeding<br>change of bowel habit<br>diarrhoea<br>constipation<br>abdominal pain<br>weight loss	internal AUC: 0.83 (0.82-0.84)<br><br>external AUC: 0.92 (0.91-0.94)	risk score	NA

Collins, 2012	UK	D+IV+EV	patients (30–84-year-old) registered with practices	CRC	2351052/ 1236601(IV)/ 2135540(EV)	age<br>family history<br>rectal bleeding<br>abdominal pain<br>appetite loss<br>weight loss<br>anaemia	internal AUC: female-0.89 (0.88–0.90); male-0.91 (0.90–0.91)<br><br>external AUC: female-0.91 (0.90–0.92); male-0.90 (0.89–0.91)	risk score	NA

Abbreviations: study design: D: model development; D+IV: model development with internal validation; D+EV: model development with external validation; AUC: area under the curve; CEA: carcinoembryonic antigen; CRC: colorectal cancer

*Five recently developed prediction models were additionally included.

Table S4: Comparison of CRC cases in SOCCS (n=1649) and LABSS (n=37)

	LABSS (n=37)	SOCCS (n=1649)	Total (n=1686)	p-value
$wGRS_{202}$*	0.10 (-0.10-0.31)	0.11 (-0.19-0.42)	0.11 (-0.19-0.42)	0.876
Age*	69.00 (59.00-77.00)	68.01 (59.33-75.36)	68.01 (59.32-75.36)	0.476
Sex				
Male	18 (48.65%)	938 (56.88%)	956 (56.70%)	0.405
Female	19 (51.35%)	711 (43.12%)	730 (43.30%)	
BMI*	25.86 (22.99-30.24)	26.11 (23.39-29.90)	26.11 (23.39-29.91)	0.925
Family history				
No	34 (91.89%)	1384 (83.93%)	1418 (84.10%)	0.279
Yes	3 (8.11%)	265 (16.07%)	268 (15.90%)	
Symptoms				
Change of bowel habit				
No	14 (37.84%)	957 (58.04%)	971 (57.59%)	0.022
Yes	23 (62.12%)	692 (41.96%)	715 (42.41%)	
Rectal bleeding				
No	20 (54.05%)	1110 (67.31%)	1130 (67.02%)	0.129
Yes	17 (45.95%)	539 (32.69%)	556 (32.98%)	
Weight loss				
No	27 (72.97%)	1410 (85.51%)	1437 (85.23%)	0.059
Yes	10 (27.03%)	239 (14.49%)	249 (14.77%)	
Anaemia				
No	24 (64.86%)	1269 (76.96%)	1293 (76.69%)	0.128
Yes	13 (35.14%)	380 (23.04%)	393 (23.31%)	
Abdominal pain				
No	31 (83.78%)	1323 (80.23%)	1354 (80.31%)	0.743
Yes	6 (16.22%)	326 (19.77%)	332 (19.69%)	

Table S5: Age-CRC restricted cubic splines

Model	R^2	Adjusted R^2	AIC	BIC
Age 3 knots	0.0760	0.0753	3438.831	3462.358
Age 4 knots	0.0760	0.0750	3440.780	3470.189
Age 5 knots	0.0773	0.0759	3439.182	3474.474
Age linear	0.0760	0.0756	3436.852	3454.498

Table S6: BMI-CRC restricted cubic splines

Model	R^2	Adjusted R^2	AIC	BIC
BMI 3 knots	0.0026	0.0018	3641.401	3664.929
BMI 4 knots	0.0027	0.0016	3642.904	3672.313
BMI 5 knots	0.0028	0.0013	3644.790	3680.082
BMI linear	0.0022	0.0018	3640.319	3657.965

Table S7: $wGRS_{202}$-CRC restricted cubic splines

Model	R^2	Adjusted R^2	AIC	BIC
wGRS 3 knots	0.0247	0.0239	3581.987	3605.515
wGRS 4 knots	0.0247	0.0236	3583.901	3613.311
wGRS 5 knots	0.0248	0.0233	3585.729	3621.020
wGRS linear	0.0244	0.0241	3580.682	3598.328

Table S8: Model C and model E comparison

Model	Method	Continuous variable approach	Case	Control	Predictors	R^2	Brier	AIC	BIC	C-statistic	Corrected C-statistic	HL (P-value)
Model C	full model	linear	1686	963	$wGRS_{202}$<br>age<br>BMI<br>sex<br>family history<br>change of bowel habits<br>rectal bleeding<br>weight loss<br>anaemia<br>abdominal pain	0.269	0.183	2915.181	2979.883	0.767 (0.749-0.786)	0.764	0.0176
Model E	full model	restricted cubic splines	1686	963	$wGRS_{202}$<br>age<br>BMI<br>sex<br>family history<br>change of bowel habits<br>rectal bleeding<br>weight loss<br>anaemia<br>abdominal pain	0.276	0.182	2914.93	3032.569	0.771 (0.753-0.790)	0.760	0.0875

Table S9: Summary of models A-D formula

Model	Formula
Model A	Model A= –1.3030 + 0.7612*wGRS + 0.0410*Age + 0.3611*Sex (M:1; F:0) –1.2411*Change of bowel habit (Yes:1; No:0) –0.6784*Abdominal pain (Yes:1; No:0).
Model B	Model B= –1.2124 + 0.0401*Age + 0.3690*Sex (M:1; F:0) –1.2411*Change of bowel habit (Yes:1; No:0) – 0.7020*Abdominal pain (Yes:1; No:0).
Model C	Model C= –07679 + 0.7603*wGRS + 0.0410*Age + 0.3631* Sex (M:1; F:0) –0.0195*BMI – 0.0024*Family history (Yes:1; No:0) – 1.2616*Change of bowel habit (Yes:1; No:0) + 0.0402*Rectal bleeding (Yes:1; No:0) – 0.0112*Weight loss (Yes:1; No:0) – 0.0531*Anaemia (Yes:1; No:0) – 0.6786*Abdominal pain (Yes:1; No:0).
Model D	Model D= –0.7170 + 0.0404*Age + 0.3714* Sex (M:1; F:0) –0.0191*BMI + 0.0349*Family history (Yes:1; No:0) – 1.2667*Change of bowel habit (Yes:1; No:0) + 0.0734*Rectal bleeding (Yes:1; No:0) – 0.0053*Weight loss (Yes:1; No:0) – 0.0661*Anaemia (Yes:1; No:0) – 0.6999*Abdominal pain (Yes:1; No:0).

Table S10: CRC risk prediction models that incorporated genetic predictors

Author, year	Study population	Model design	Study design	Sample size	Outcome	Type of genetic variants used	GRS computation	Non-genetic factors included in the model	C-statistic of model without genetic predictors	C-statistic of genetic predictors-enhanced model	IDI; NRI
Abe, 2017	Japanese	D+EV	CC	derivation: 558 cases/ 1116 controls<br><br>replication: 547 cases/ 547 controls	CRC	11 SNPs	unweighted GRS	NA	NA	derivation: 0.639<br><br>replication: 0.569	NA
Balavarca, 2019	German	D	CC	291 CRC cases/ 487 controls	CRC	39 SNPs	unweighted GRS<br><br>wGRS (weights derived from the same study)	age, sex, family history, smoking, alcohol, red meat, NSAIDs, previous colonoscopy and polyps history	0.584 (0.545-0.622)	unweighted GRS: 0.636 (0.599–0.672);<br><br>wGRS: 0.616 (0.579–0.654)	NA
Chandler, 2018	US	D	cohort	23,294 study population/ 329 cases	CRC	5 SNPs	unweighted GRS	NA	NA	NA	NA
Cho, 2019	Korean	D	CC	632 cases/ 1295 controls	CRC	13 SNPs	unweighted GRS<br><br>wGRS (weights derived from the same study)	BMI, physical activity, diet, smoking, alcohol	NA	NA	NA
deKort S, 2019	Dutch	D	CC	1907 cases/ 2729 controls	CRC	18 SNPs	unweighted GRS	age, BMI, pant size, CRC first degree relative, smoking, physical activity, alcohol, meat, vegetables, fish, sweets, added sugar, saturated fats and fiber, total energy.	NA	NA	NA
Dunlop, 2013	European descendants	D+IV+EV (CV)	CC	genotypes alone: 39,266; In combination with other factors: 11,324;<br><br>external validation case-control sets: 1563 Swedish cases and 1504	CRC	10 SNPs	unweighted GRS	age, sex, family history	0.57	0.59	NA

				controls, 702 Finnish cases and 418 controls.							
Hiraki, 2013	European descendants	D	CC	10,061 cases/ 12,768 controls	CRC	4 SNPs	unweighted GRS	age, sex, smoking, BMI, family history, NSAIDs, alcohol, dietary calcium, folate and red meat, sedentary status, hormone replacement therapy	NA	NA	NA
Hosono, 2016	Japanese	D+EV	CC	derivation: 558 cases/ 1116 controls<br><br>replication: 547 cases/ 547 controls	CRC	6 SNPs	unweighted GRS	age, smoking, alcohol, folate intake, BMI, family history, physical activity	derivation: 0.701<br><br>replication: 0.523	derivation: GRS-0.605; GRS+non-genetic predictors-0.717<br><br>replication: GRS-0.639; GRS+non-genetic predictors-0.636	NA
HsuL, 2015	European descendants	D+IV+EV (bootstrap)	CC	training: 5811 cases/ 6302 controls<br><br>validation: 866 cases/ 869 controls	CRC	27 SNPs	unweighted GRS<br><br>wGRS (weights derived from the literature)	age, sex, family history, history of endoscopic examinations	men: 0.51 (0.48-0.53)<br><br>women: 0.52 (0.5-0.55)	men: 0.59 (0.54-0.64)<br><br>women: 0.56 (0.51-0.61)	NA
Huyghe, 2019	European descendants	D	CC	1439 cases/ 720 controls	CRC	95 SNPs	wGRS (weights derived from the same study)	NA	NA	NA	NA
Ibáñez-Sanz, 2017	Spanish	D+IV (CV)	CC	1336 cases/ 2744 controls	CRC	21 SNPs	unweighted GRS<br><br>wGRS (weights derived from the literature and from the same study)	alcohol, BMI, physical activity, red meat and vegetables, NSAISs/aspirin, family history	0.61 (0.59-0.64)	0.63 (0.60-0.66)	NA
Iwasaki, 2017	Japanese	D+IV (CV)	CC	675 cases/ 675 controls	CRC	6 SNPs	wGRS (weights derived from the same study)	age, BMI, alcohol, smoking	0.60	0.66	IDI (0.0052; 95% CI: 0.0023– 0.0081)<br><br>continuous NRI (0.36; 95% CI: 0.0023–0.71), and NRI (0.26; 95% CI: 0.0039–0.43)

Jenkins, 2019	North American; Australian	D	CC	1181 cases/ 999 controls	CRC	45 SNPs	wGRS (weights derived from the literature)	family history	NA	NA	NA
Jeon, 2018	European descendants	D+IV (bootstrap)	CC	training: 4875 cases/ 5291 controls<br><br>validation: 4873 cases/ 5299 controls	CRC	63 SNPs	wGRS (weights derived from the same study)	sex, BMI, education, type 2 diabetes, smoking, alcohol, NSAID/ aspirin use, regular use of postmenopausal hormones, dietary factors, total-energy, and physical activity	men: 0.60 (0.59–0.61);<br><br>women: 0.60 (0.59–0.61)	men: 0.63 (0.62–0.64);<br><br>women: 0.62 (0.61–0.63)	NA
Jo, 2012	Korean	D+IV (bootstrap)	CC	187 cases/ 976 controls	CRC	3 SNPs: men<br><br>5 SNPs: women	unweighted GRS<br><br>wGRS (weights derived from the same study)	family history, age	men: 0.692 (0.647– 0.732);<br><br>women: 0.603 (0.569–0.637)	GRS:<br>men: 0.720 (0.682– 0.767);<br>women: 0.650 (0.615–0.680)<br><br>wGRS:<br>men: 0.719 (0.677– 0.761);<br>women: 0.646 (0.612–0.674)	NA
Jung, 2015	Korean	D	CC	173 cases/ 1514 controls	CRC	7 SNPs	unweighted GRS<br><br>wGRS (weights derived from the same study)	age, sex, smoking, fasting serum glucose, family history	0.73 (0.69-0.78)	0.74 (0.70-0.78)	NRI: 0.17 (-0.05-0.37)
Jung, 2019	European descendants	D+IV (bootstrap)	cohort	6539 study population/ 472 cases	CRC	54 SNPs	NA	age, % calories from saturated fatty acid	NA	NA	NA
Marshall, 2010	North American	D+IV (bootstrap)	CC	training: 112 cases/ 120 controls<br><br>validation: 202 cases/ 208 controls	CRC	7 genes (ANXA3, CLEC4D, LMNB1, PRRG4, TNFAIP6, VNN1 and IL2RB)	NA	NA	NA	training: 0.80 (0.74-0.85)<br><br>validation: 0.80 (0.76-0.84)	NA
Prizment, 2013	Caucasian	D	cohort	8657 study population/ 205 cases	CRC	20 SNPs	wGRS (weights derived from the literature)	NA	NA	NA	NA

Rodriguez-Broadbent, 2017	European descendants	D	CC	9254 cases/ 18,386 controls	CRC	total cholesterol (n=38); triglyceride (n=14); LDL (n=9); HDL (n=43)	NA	NA	NA	NA	NA
Schmit, 2019	European descendants	D+EV	CC	Discovery stage: 36,948 cases and 30,864 controls; Replication set: 12,952 cases and 48,383 controls; Generalizability in East Asians, African Ameri-cans, and Hispanics: 12,085 cases and 22,083 controls.	CRC	76 SNPs	wGRS (weights derived from the same study)	NA	NA	NA	NA
Shi, 2019	Caucasian	D	CC	387 cases/ 13,427 controls	CRC	30 SNPs	wGRS (weights derived from the literature)	NA	NA	NA	NA
Smith, 2018	UK	D	cohort	Taylor model: 361,543 (1623 cases); Wells model: 286,877 (1294 cases)	CRC	41 SNPs	wGRS (weights derived from the literature)	Taylor model: age-specific CRC rates and estimated RR for different degrees of FH of CRC. Wells model: age, diabetes, multi-vitamin usage, FH of CRC, education, BMI, alcohol use, physical activity, NSAIDs, red meat intake, smoking and estrogen use (women only).	Taylor model: 0.67 (0.65–0.68); Wells model: 0.68 (0.67–69)	Taylor model:0.69 (0.67–0.70); Wells model: 0.69 (0.65–0.68)	NA

Thrift, 2015	European descendants	D	CC	10,226 cases/ 10,286 controls	CRC	696 SNPs	wGRS (weights derived from the literature)	NA	NA	NA	NA
Thrift, 2015	European descendants	D	CC	10,226 cases/ 10,286 controls	CRC	77 SNPs: BMI<br><br>47 SNPs: waist-hip ratio	wGRS (weights derived from the literature)	NA	NA	NA	NA
Wang, 2013	Chinese	D+IV (CV)	CC	218 cases/ 385 controls	CRC	16 SNPs: short model;<br><br>26 SNPs: full model	NA	NA	NA	16-SNPs: 0.724<br><br>26-SNPs: 0.734	NA
Wang, 2018	Chinese	D	cohort	64 cases/ 9636 controls	CRC	9 SNPs	NA	AFP, CA19-9, CEA	AFP level: 0.523 (0.456–0.591);<br><br>CA19–9 level:0.524 (0.451– 0.597);<br><br>CEA level: 0.568 (0.492–0.645);<br><br>AFP, CA19–9, CEA level: 0.509 (0.439–0.579)	AFP level - genetic corrected: 0.524 (0.458–0.591);<br><br>CA19–genetic corrected level: 0.525 (0.452–0.597);<br><br>CEA level-genetic corrected 0.572 (0.495–0.649);<br><br>AFP, CA19–9, CEA level-genetic corrected: 0.564 (0.487–0.641)	NA
Weigl, 2018	German	D	CC	genotype: 294 advanced neoplasms, 249 non-advanced adenomas, 500 controls<br><br>replication: 462 controls, 140 advanced adenomas, 355	CRC	48 SNPs (replication analyses within the TCPS with a subset of 35 SNPs of the original GRS)	unweighted GRS;<br><br>wGRS (weights derived from literature)	age, sex, previous colonoscopy, physical activity, BMI	model adjusted for age and gender: 0.599;<br><br>model adjusted for age, gender, previous colonoscopy, physical activity: 0.607;	model adjusted for age and gender: 0.653;<br><br>model adjusted for age, gender, previous colonoscopy, physical activity: 0.658;	NRI and IDI of model including GRS were respectively of 0.29 (0.14–0.43) and 0.04 (0.03–0.05) when the model was adjusted for age and gender; 0.30 (0.15–0.44) and 0.04 (0.03–0.05) when adjusted for age, gender, previous

				non-advanced adenomas					model adjusted for age, gender, previous colonoscopy, physical activity, BMI: 0.615	model adjusted for age, gender, previous colonoscopy, physical activity, BMI: 0.665	colonoscopy, physical activity and 0.29 (0.14–0.43) and 0.04 (0.03–0.05) when the model was adjusted for age, gender, previous colonoscopy, physical activity, BMI.
Weigl, 2018	German	D	CC	2363 cases/ 2198 controls	CRC	44 SNPs	unweighted GRS<br><br>wGRS (weights derived from the literature)	age, sex, education, previous colonoscopy, smoking, hormone replacement therapy, BMI, family history	NA	NA	NA
Xin, 2018	Chinese	D+IV (CV)	CC	1316 cases/ 2229 controls	CRC	14 SNPs	unweighted GRS<br><br>wGRS (weights derived from the literature and from the same study)	smoking	OR (95%CI) of: 2.70 (2.06–3.54) in the simple count GRS model, 2.74 (2.19–3.43) in the directed logistic regression GRS model, 2.56 (2.05– 3.20) in the odds ratio weighted GRS model, 2.90 (2.32–3.63) in the explained variance weighted GRS model, 2.51 (2.01–3.14) in the explained variance weighted OR GRS model.	NA	simple-count-GRS vs. logistic regression weighted OR-GRS showed an NRI of – 0.082 (− 0.159, − 0.007; p value: 0.033) and an IDI of – 0.002 (− 0.004,− 2.33E− 04; 0.028); the simple-count- GRS vs. explained variance weighted OR-GRS showed an NRI of 0.017 (− 0.055, 0.090; 0.638) and an IDI of 2.80E− 04 (− 0.001, 0.001; 0.567); logistic regression weighted-GRS vs. explained variance weighted OR-GRS showed an NRI – 0.077 (− 0.153, − 0.001; 0.046) and an IDI of − 5.54E− 04 (− 0.001, − 3.17E− 05; 0.038). In addition, a model including only smoking factors was with a model including smoking factors and simple count GRS (SC-GRS), with an increased AUC, NRI

											and IDI in combined model of 0.084, 0.317 (0.225, 0.408) and 0.031 (0.023, 0.039)
Xin, 2019	Chinese	D+IV (CV)	CC	Chinese study: 2248 cases/ 3173 controls;<br><br>GECCO study: 4461 cases/ 4140 controls	CRC	Chinese study: 19 SNPs vs. 58 SNPs<br><br>GECCO study: 19 SNPs vs. 75 SNPs	wGRS (weights derived from the same study)	sex, age, first principal component	NA	Chinese studies:19 SNPs model of 0.597 (0.581– 0.613), 58 SNPs model of 0.623 (0.604–0.642);<br><br>GECCO study:19 SNPs model of 0.575 (0.563–0.587), 58 SNPs model of 0.585 (0.573–0.597)	NA
Yeh, 2007	Chinese	D	CC	727 cases/ 736 controls	CRC	10 SNPs	NA	age, education, physical activity, coffee, cigarette consumption, alcohol, staple consumption, meat, vegetable/ fruit and fish/ shrimp intake.	NA	NA	NA
Zhang, 2017	Chinese	D	CC	369 cases/ 929 controls	CRC	4 SNPs	NA	age, BMI, physical activity, emotion status, mental stress, cholesterol, drinking and smoking, vegetables and seafood consumption	NA	NA	NA

Table S10 reproduced from Sassano et al., 2022.

Abbreviations: study design: D: model development; D+IV: model development with internal validation; D+EV: model development with external validation; AUC: area under the curve; CC: case-control; CEA: carcinoembryonic antigen; CRC: colorectal cancer; CV: cross-validation; NA: non-applicable; TRS: traditional risk score

Table S11: Methods for variable selection in the development of the final prediction model

Method	Characteristics	Strengths	Limitations
Full model	All candidate variables are included in the final model.<br><br>Candidate variables are determined by the authors based on clinical knowledge and previous literature.	• Less vulnerable to model overfitting, selection bias, and dataset-specific problems.<br><br>• Computationally simple.<br><br>• The full model with a limited number of important predictors may have better performance.	• Requires reliable prior knowledge about what variables are predictive.<br><br>• It is not practical to include a large set of variables in a model. The full model may exceed the number of dimensions the dataset can reasonably support.<br><br>• Including too many variables in a model may lead to a close fit to the data, and the relationship between variables and the predicted outcome that exists in the particular dataset, but not in the true population.<br><br>• The full model is not always easy to define.
LASSO (penalization)	The less important predictors of the dataset are penalized by the lasso regression. Imposes a "penalty" to bias predictor coefficients toward zero for not being selected for the final model.<br><br>The penalty is a function of the coefficient's absolute magnitude and a weight, λ.	• Does not require reliable prior knowledge about what variables are predictive.<br><br>• Useful to identify a small set of the most influential predictors in the dataset with many candidate variables.<br><br>• Simplifies and automates the process of selecting predictors to those most influential to predictive accuracy.<br><br>• If correctly weighted, can enhance prediction accuracy.<br><br>• Produces a simple model with fewer predictors which is preferred over a complex model with many predictors. The simple model is easier to interpret, generalise, and use in practice.	• May exclude predictors that have moderate predictive power.<br><br>• May include implausible predictors and omit the known predictors which are verified in the previous literature.<br><br>• Individual predictors are difficult to interpret because the focus is on the best-combined prediction rather than the accuracy of the estimation and interpretation of the contribution of individual predictors.<br><br>• Computationally intensive.

Table S12: Random forest model F and model G comparison

Model	Method	Training/Test	Case	Control	mtry	No.tree	Predictors	OBB_error rate	C-statistic	Sensitivity	Specificity	Positive predictive value	Negative predictive value
Model F	random forest	training	1181	675	3	500	$wGRS_{202}$<br>age<br>BMI<br>sex<br>family history<br>change of bowel habits<br>rectal bleeding<br>weight loss<br>anaemia<br>abdominal pain	27.64%	0.986 (0.979-0.990)	0.972	0.993	0.988	0.984
Model F	random forest	test	505	288	3	500	$wGRS_{202}$<br>age<br>BMI<br>sex<br>family history<br>change of bowel habits<br>rectal bleeding<br>weight loss<br>anaemia<br>abdominal pain	27.64%	0.754 (0.723-0.784)	0.569	0.859	0.698	0.778
Model G	random forest	training	1181	675	3	500	age<br>BMI<br>sex<br>family history<br>change of bowel habits<br>rectal bleeding<br>weight loss<br>anaemia<br>abdominal pain	27.37%	0.903 (0.889-0.916)	0.807	0.958	0.916	0.897
Model G	random forest	test	505	288	3	500	age<br>BMI<br>sex<br>family history<br>change of bowel habits<br>rectal bleeding<br>weight loss<br>anaemia<br>abdominal pain	27.37%	0.740 (0.708-0.770)	0.549	0.850	0.675	0.767

The random forest regression models were built on the training sets (70%) and validated in the test set (30%). OBB_error rate: out-of-bag estimate (a method of measuring the prediction error of random forests, boosted decision trees).

Figure S1: Flow chart for $wGRS_{137}$, $wGRS_{163}$, $wGRS_{202}$

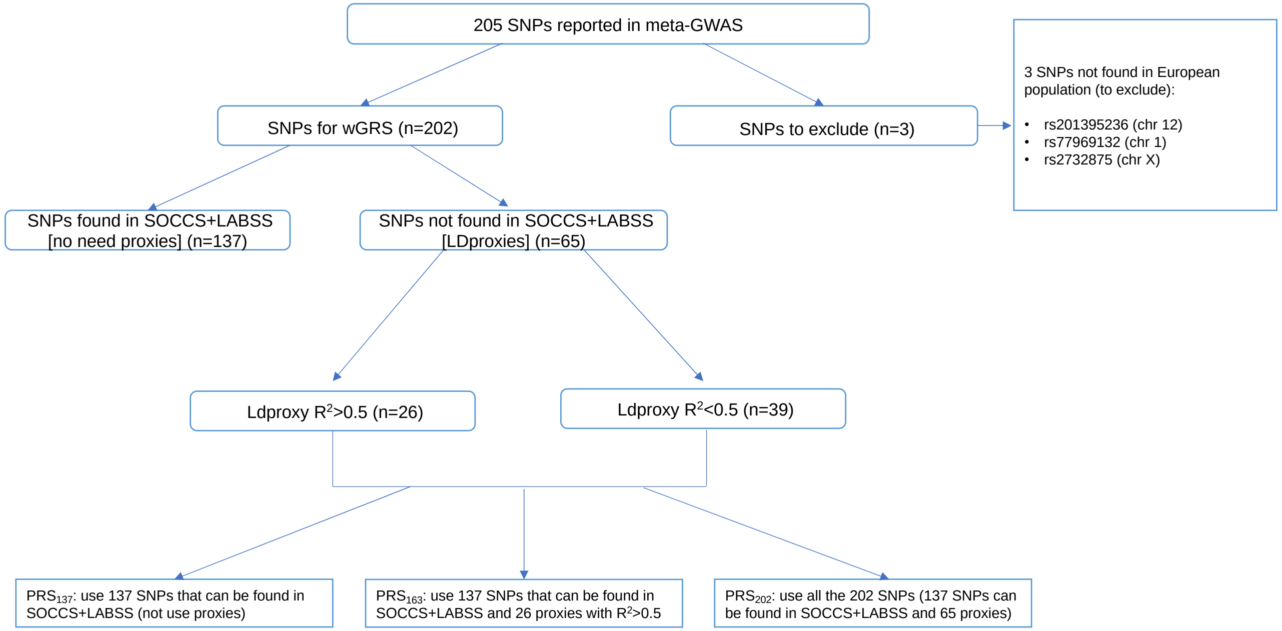

Figure S2: TRIPOD checklist

Section/Topic	Item		Checklist Item	Page
Title and abstract				
Title	1	D;V	Identify the study as developing and/or validating a multivariable prediction model, the target population, and the outcome to be predicted	
Abstract	2	D;V	Provide a summary of objectives, study design, setting, participants, sample size, predictors, outcome, statistical analysis, results, and conclusions	
Introduction				
Background and objectives	3a	D;V	Explain the medical context (including whether diagnostic or prognostic) and rationale for developing or validating the multivariable prediction model, including references to existing models	
	3b	D;V	Specify the objectives, including whether the study describes the development or validation of the model or both	
Methods				
Source of data	4a	D;V	Describe the study design or source of data (eg, randomized trial, cohort, or registry data), separately for the development and validation data sets, if applicable	
	4b	D;V	Specify the key study dates, including start of accrual; end of accrual; and if applicable, end of follow-up	
Participants	5a	D;V	Specify key elements of the study setting (eg, primary care, secondary care, general population) including number and location of centers	
	5b	D;V	Describe eligibility criteria for participants	
	5c	D;V	Give details of treatments received, if relevant	
Outcome	6a	D;V	Clearly define the outcome that is predicted by the prediction model, including how and when assessed	
	6b	D;V	Report any actions to blind assessment of the outcome to be predicted	
Predictors	7a	D;V	Clearly define all predictors used in developing the multivariable prediction model, including how and when they were measured	
	7b	D;V	Report any actions to blind assessment of predictors for the outcome and other predictors	
Sample size	8	D;V	Explain how the study size was arrived at	
Missing data	9	D;V	Describe how missing data were handled (eg, complete-case analysis, single imputation, multiple imputation) with details of any imputation method	
Statistical analysis methods	10a	D	Describe how predictors were handled in the analyses	
	10b	D	Specify type of model, all model-building procedures (including any predictor selection), and method for internal validation	
	10c	V	For validation, describe how the predictions were calculated	
	10d	D;V	Specify all measures used to assess model performance and, if relevant, to compare multiple models	
	10e	V	Describe any model updating (eg, recalibration) arising from the validation, if done	
Risk groups	11	D;V	Provide details on how risk groups were created, if done	
Development vs. validation	12	V	For validation, identify any differences from the development data in setting, eligibility criteria, outcome, and predictors	
Results				
Participants	13a	D;V	Describe the flow of participants through the study, including the number of participants with and without the outcome and, if applicable, a summary of the follow-up time. A diagram may be helpful.	
	13b	D;V	Describe the characteristics of the participants (basic demographics, clinical features, available predictors), including the number of participants with missing data for predictors and outcome	
	13c	V	For validation, show a comparison with the development data of the distribution of important variables (demographics, predictors, and outcome)	
Model development	14a	D	Specify the number of participants and outcome events in each analysis	
	14b	D	If done, report the unadjusted association between each candidate predictor and outcome	
Model specification	15a	D	Present the full prediction model to allow predictions for individuals (ie, all regression coefficients, and model intercept or baseline survival at a given time point)	
	15b	D	Explain how to use the prediction model	
Model performance	16	D;V	Report performance measures (with CIs) for the prediction model	
Model updating	17	V	If done, report the results from any model updating (ie, model specification, model performance)	
Discussion				
Limitations	18	D;V	Discuss any limitations of the study (such as nonrepresentative sample, few events per predictor, missing data)	
Interpretation	19a	V	For validation, discuss the results with reference to performance in the development data, and any other validation data	
	19b	D;V	Give an overall interpretation of the results, considering objectives, limitations, results from similar studies, and other relevant evidence	
Implications	20	D;V	Discuss the potential clinical use of the model and implications for future research	
Other information				
Supplementary information	21	D;V	Provide information about the availability of supplementary resources, such as study protocol, Web calculator, and data sets	
Funding	22	D;V	Give the source of funding and the role of the funders for the present study	

*Items relevant only to the development of a prediction model are denoted by D, items relating solely to a validation of a prediction model are denoted by V, and items relating to both are denoted D;V. We recommend using the TRIPOD Checklist in conjunction with the TRIPOD Explanation and Elaboration document.

(Source: adapted from https://www.tripod-statement.org/ with permission covered by TRIPOD, open access). D: model development; V: model validation.

Figure S3: Plot-association between age and risk of CRC

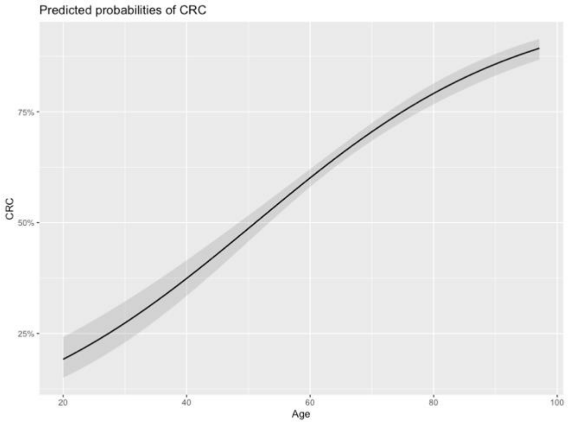

[Y-axis presents CRC probability rate. X-axis presents age. The black solid line represents the crude effect. The shaded regions show 95% CIs.]

Figure S4: Plot-association between BMI and risk of CRC

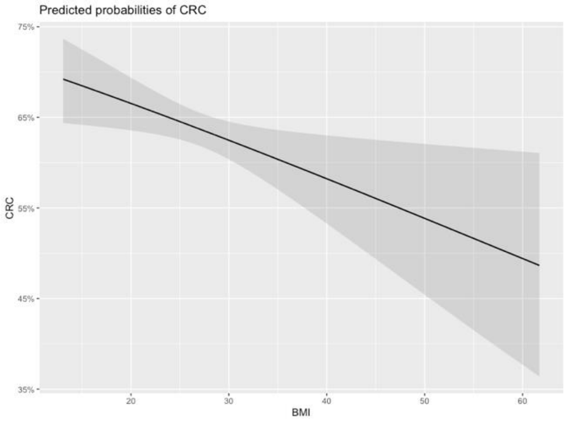

[Y-axis presents CRC probability rate. X-axis presents BMI. The black solid line represents the crude effect. The shaded regions show 95% CIs.]

Figure S5: Plot-association between $wGRS_{202}$ and risk of CRC

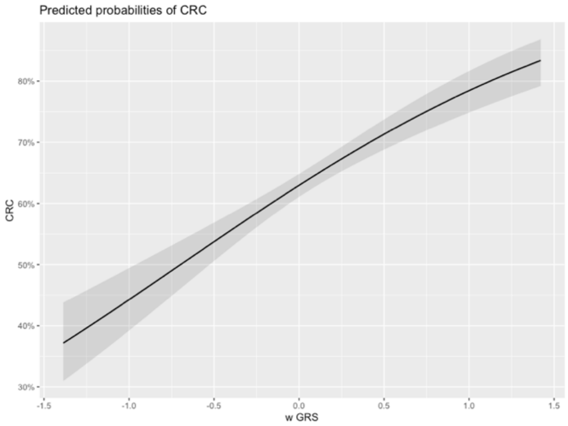

[Y-axis presents CRC probability rate. X-axis presents wGRS202. The black solid line represents the crude effect. The shaded regions show 95% CIs.]

Figure S6: Restricted cubic splines fit age with CRC risk

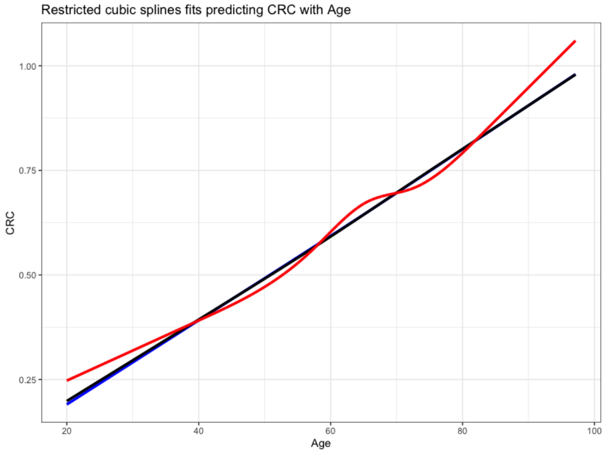

[Y-axis presents CRC probability rate. X-axis presents age. The blue solid line is restricted cubic splines using three knots. The black solid line is restricted cubic splines using four knots. The red solid line is restricted cubic splines using five knots.]

Figure S7: Restricted cubic splines fit BMI with CRC risk

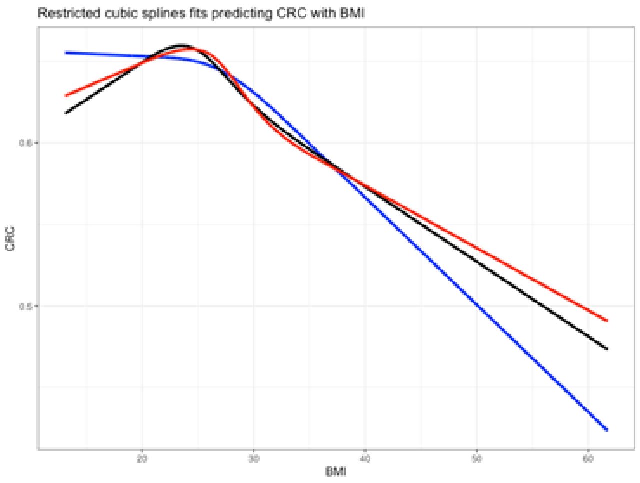

[Y-axis presents CRC probability rate. X-axis presents BMI. The blue solid line is restricted cubic splines using three knots. The black solid line is restricted cubic splines using four knots. The red solid line is restricted cubic splines using five knots.]

Figure S8: Restricted cubic splines fit $wGRS_{202}$ with CRC risk

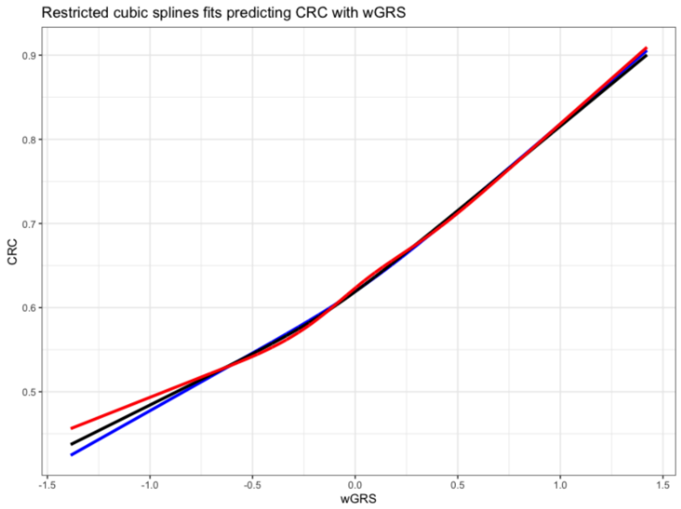

[Y-axis presents CRC probability rate. X-axis presents wGRS202. The blue solid line is restricted cubic splines using three knots. The black solid line is restricted cubic splines using four knots. The red solid line is restricted cubic splines using five knots.]

Figure S9: ROC curves- wGRS$_{137}$, wGRS$_{163}$, wGRS$_{202}$ comparison

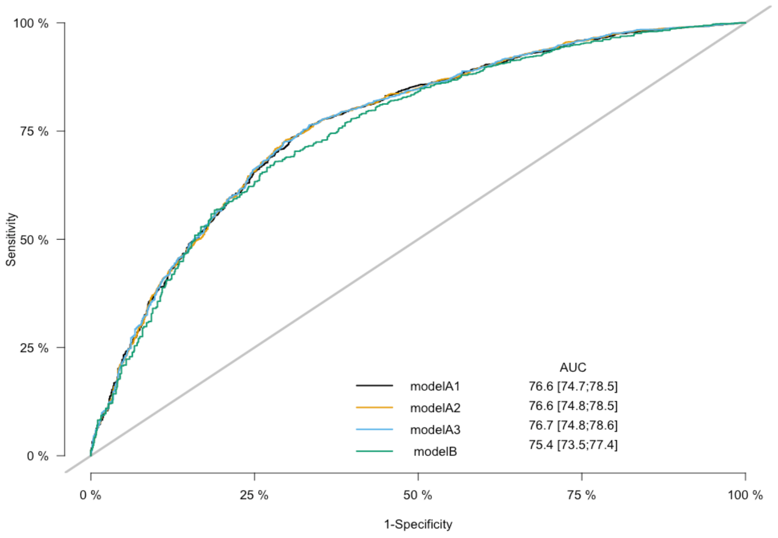

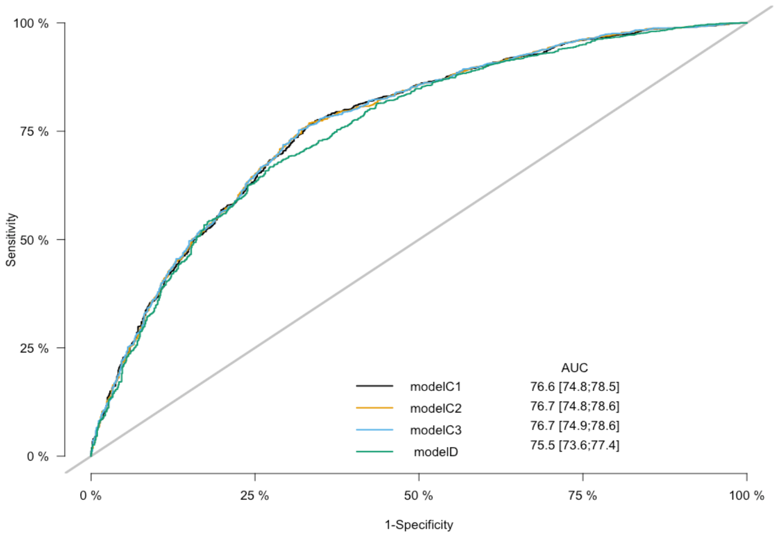

[LASSO model: model A1=wGRS$_{137}$; model A2=wGRS$_{163}$; model A3=wGRS$_{202}$]

[model A1 vs A2: P=0.2744; model A1 vs A3: P=0.2061; model A2 vs A3: P=0.5580]

[Full model: model C1=wGRS$_{137}$; model C2=wGRS$_{163}$; model C3=wGRS$_{202}$]

[model C1 vs C2: P=0.2344; model C1 vs C3: P=0.1664; model C2 vs C3: P=0.4496]

Figure S10: Calibration curves- wGRS$_{137}$, wGRS$_{163}$, wGRS$_{202}$ comparison

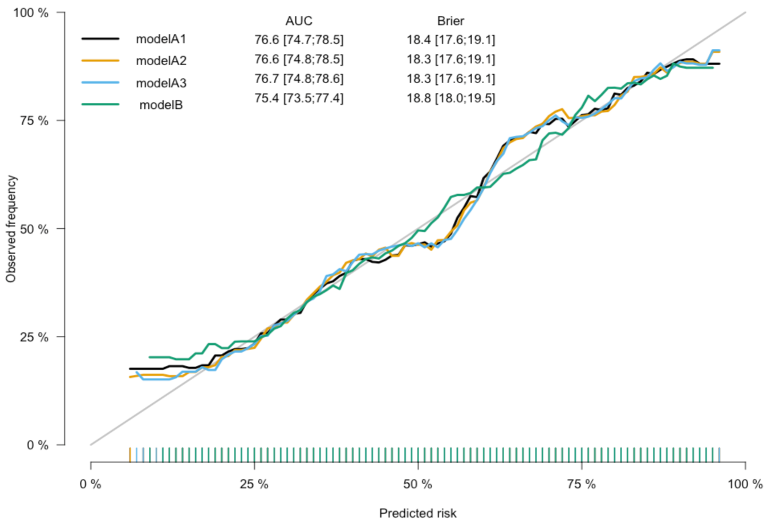

[LASSO model: model A1=wGRS$_{137}$; model A2=wGRS$_{163}$; model A3=wGRS$_{202}$]

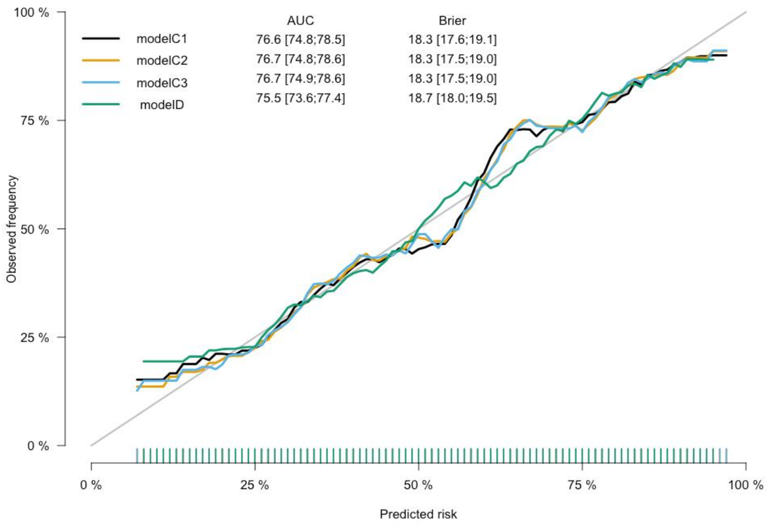

[Full model: model C1=wGRS$_{137}$; model C2=wGRS$_{163}$; model C3=wGRS$_{202}$]

Figure S11: Random forest parameters tuning: mtry versus OOB error

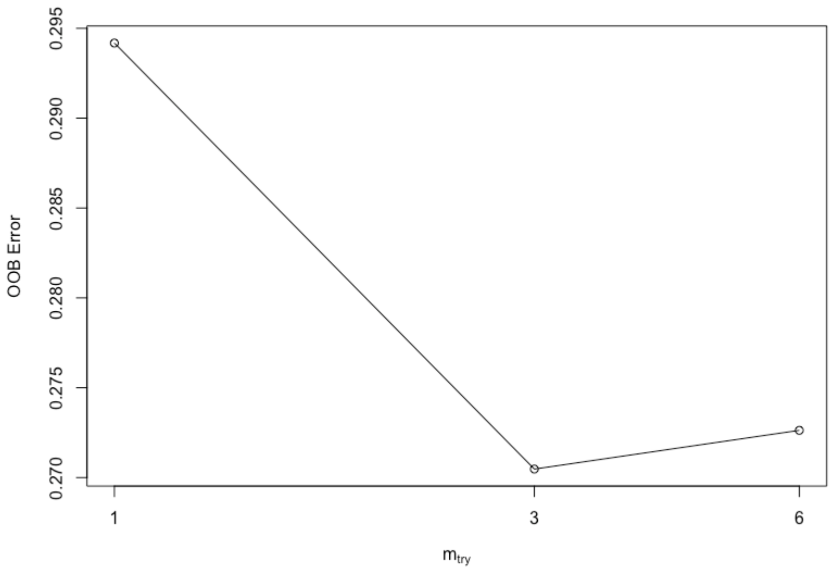

[a tree is grown mvariables (mtry) areselected at random from all nvariables (mtry ≤n) and the best split of all mtry is used at each node. mtry=3, OOB error rate lowest=26.01%]

Figure S12: Model F_ Plot of OOB errors against number of trees

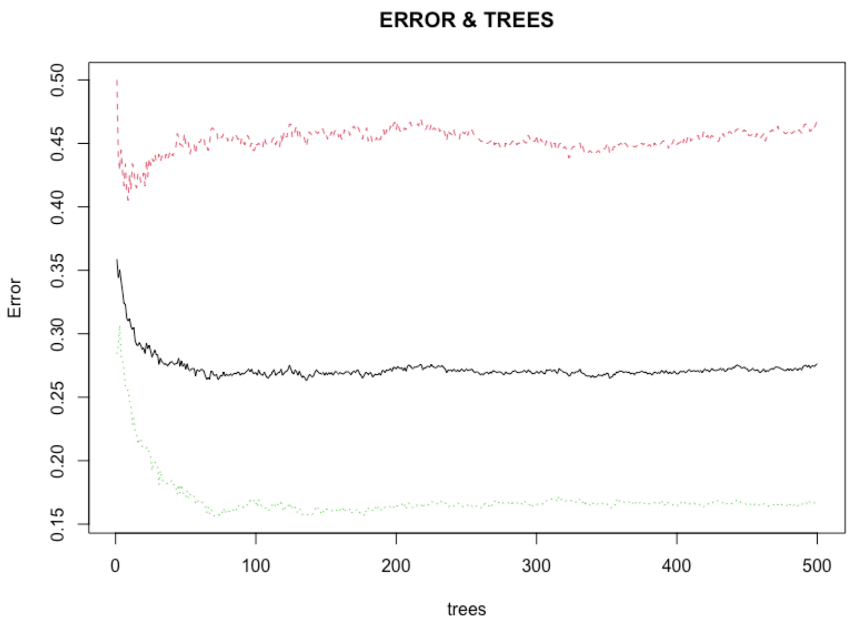

[black: overall relative error rates; red: control relative error rates; greed: case relative error rates]

Figure S13: Model G_ Plot of OOB errors against number of trees

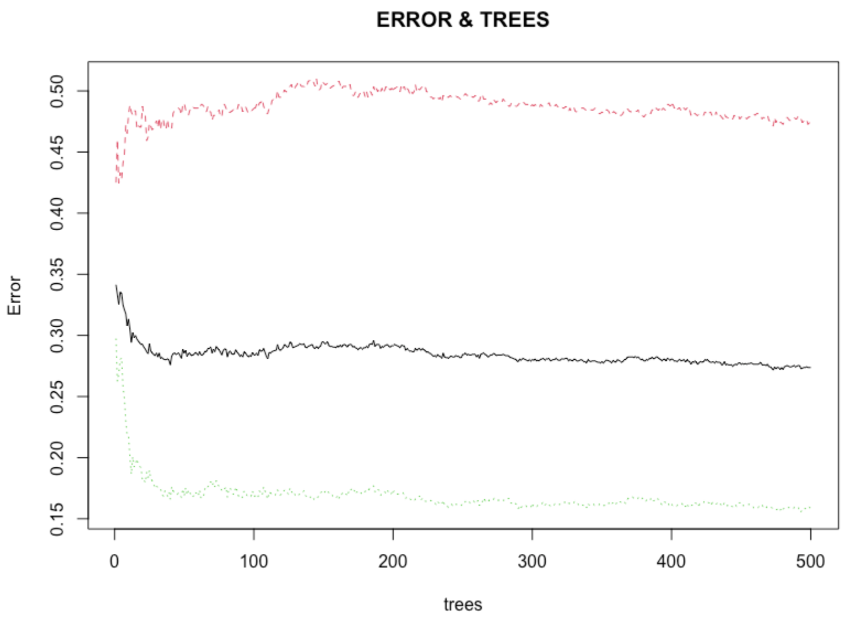

[black: overall relative error rates; red: control relative error rates; greed: case relative error rates]
